# Risk of heart failure in elderly patients with atrial fibrillation and diabetes taking different oral anticoagulants: a nationwide cohort study

**DOI:** 10.1186/s12933-022-01688-1

**Published:** 2023-01-06

**Authors:** Shu-Man Lin, Peter Pin-Sung Liu, Yu-Kang Tu, Edward Chia-Cheng Lai, Jih-I Yeh, Jin-Yi Hsu, Kashif M. Munir, Carol Chiung-Hui Peng, Huei-Kai Huang, Ching-Hui Loh

**Affiliations:** 1 Department of Physical Medicine and Rehabilitation, Hualien Tzu Chi Hospital, Buddhist Tzu Chi Medical Foundation, Hualien, Taiwan; 2 School of Medicine, Tzu Chi University, Hualien, Taiwan; 3 Center for Aging and Health, Hualien Tzu Chi Hospital, Buddhist Tzu Chi Medical Foundation, No. 707, Sec. 3, Chung Yang Rd., Hualien, 97002 Taiwan; 4 Institute of Medical Sciences, Tzu Chi University, Hualien, Taiwan; 5 Institute of Epidemiology and Preventive Medicine, College of Public Health, National Taiwan University, Taipei, Taiwan; 6 Department of Dentistry, National Taiwan University Hospital and School of Dentistry, National Taiwan University, Taipei, Taiwan; 7 School of Pharmacy, Institute of Clinical Pharmacy and Pharmaceutical Sciences, College of Medicine, National Cheng Kung University, Tainan, Taiwan; 8 Department of Family Medicine, Hualien Tzu Chi Hospital, Buddhist Tzu Chi Medical Foundation, No. 707, Sec. 3, Chung Yang Rd., Hualien, 97002 Taiwan; 9 Division of Endocrinology, Diabetes and Nutrition, University of Maryland School of Medicine, Baltimore, MD USA; 10 Section of Endocrinology, Diabetes, Nutrition & Weight Management, Boston University School of Medicine, Boston, MA USA

**Keywords:** Oral anticoagulant, Heart failure, Atrial fibrillation, Diabetes mellitus, Elderly

## Abstract

**Background:**

Heart failure (HF) is a critical complication in elderly patients with atrial fibrillation (AF) and diabetes mellitus (DM). Recent preclinical studies suggested that non-vitamin K antagonist oral anticoagulants (NOACs) can potentially suppress the progression of cardiac fibrosis and ischemic cardiomyopathy. Whether different oral anticoagulants influence the risk of HF in older adults with AF and DM is unknown. This study aimed to evaluate the risk of HF in elderly patients with AF and DM who were administered NOACs or warfarin.

**Methods:**

A nationwide retrospective cohort study was conducted based on claims data from the entire Taiwanese population. Target trial emulation design was applied to strengthen causal inference using observational data. Patients aged  ≥ 65 years with AF and DM on NOAC or warfarin treatment between 2012 and 2019 were included and followed up until 2020. The primary outcome was newly diagnosed HF. Propensity score-based fine stratification weightings were used to balance patient characteristics between NOAC and warfarin groups. Hazard ratios (HRs) were estimated using Cox proportional hazard models.

**Results:**

The study included a total of 24,835 individuals (19,710 NOAC and 5,125 warfarin users). Patients taking NOACs had a significantly lower risk of HF than those taking warfarin (HR = 0.80, 95% CI 0.74–0.86, p < 0.001). Subgroup analyses for individual NOACs suggested that dabigatran (HR = 0.86, 95% CI 0.80–0.93, p < 0.001), rivaroxaban (HR = 0.80, 95% CI 0.74–0.86, p < 0.001), apixaban (HR = 0.78, 95% CI 0.68–0.90, p < 0.001), and edoxaban (HR = 0.72, 95% CI 0.60–0.86, p < 0.001) were associated with lower risks of HF than warfarin. The findings were consistent regardless of age and sex subgroups and were more prominent in those with high medication possession ratios. Several sensitivity analyses further supported the robustness of our findings.

**Conclusions:**

This nationwide cohort study demonstrated that elderly patients with AF and DM taking NOACs had a lower risk of incident HF than those taking warfarin. Our findings suggested that NOACs may be the preferred oral anticoagulant treatment when considering the prevention of heart failure in this vulnerable population. Future research is warranted to elucidate causation and investigate the underlying mechanisms.

**Supplementary Information:**

The online version contains supplementary material available at 10.1186/s12933-022-01688-1.

## Background

In the elderly population, atrial fibrillation (AF) and diabetes mellitus (DM) are both global epidemics and important public health problems [1, 2]. Due to their high prevalence and incidence, these two chronic conditions commonly coexist. Heart failure (HF), another global epidemic, affects at least 26 million people worldwide and is one of the leading causes of morbidity, hospitalization, and mortality in older adults, placing a huge financial burden on the health care system [3, 4]. Current evidence indicates that HF is a critical complication in patients with AF and DM. Hyperglycemia, insulin resistance, and hyperinsulinemia in DM can trigger a cascade of deleterious effects contributing to development of HF and effort intolerance [5–7]. The tachycardia, irregularity, loss of atrial systole, and cardiac fibrosis in patients with AF also contribute to HF development [4, 8]. Since AF, DM, and aging are all major risk factors of HF [4, 5, 9] and concomitant HF in elderly patients with AF and DM could increase their risk of stroke, worsen patient prognoses, and increase the healthcare cost burden [5, 10, 11], the prevention of HF development in the elderly population with AF and DM is crucial.

Long-term oral anticoagulant treatment is an essential medication for stroke prevention in elderly patients with AF and DM [12, 13]. Warfarin, a vitamin K antagonist, has been used to prevent stroke for decades. Non-vitamin K antagonist oral anticoagulants (NOACs) have been approved as another choice of oral anticoagulants and have been found to offer comparable efficacy and safety for stroke prevention [14]. In addition to its anticoagulation effect, recent studies have suggested that NOACs, compared with warfarin, were linked to better glycemic control and lower diabetes complication risks [15–17]. Additionally, recent preclinical studies suggested that NOACs have potential anti-inflammatory effects and may suppress the progression of cardiac fibrosis and ischemic cardiomyopathy, all of which are related to the pathophysiology of HF [18–20]. Thus, it is reasonable to suppose that NOACs may have a beneficial effect on preventing HF compared with warfarin in patients with AF and DM. As HF is also an independent and potent risk factor for stroke development [11], of which oral anticoagulants are mainly prescribed for prevention, choosing appropriate oral anticoagulant types to decrease incident HF risks is crucial.

However, to date, the evidence comparing the risk of HF between NOAC and warfarin use is still lacking, even though this issue is critical for improving patient prognosis in elderly adults already with AF and DM. Therefore, we used nationwide cohort data to investigate the risk of HF development in elderly patients with AF and DM taking NOAC versus warfarin.

## Methods

### Data sources

We conducted a nationwide retrospective cohort study using data from the National Health Insurance Research Database (NHIRD) in Taiwan. The National Health Insurance program, a mandatory single-payer program administered by Taiwan’s government, covered more than 99% of the entire population in Taiwan (approximately 23.6 million individuals) [21, 22]. The NHIRD contains patient demographic information and medical claims for all inpatient, outpatient, and emergency care services in Taiwan. The diagnostic and procedure codes in NHIRD were derived using the International Classification of Diseases, Ninth Revision, Clinical Modification (ICD-9-CM) codes before 2016 and the International Classification of Diseases, Tenth Revision, Clinical Modification (ICD-10-CM) codes since 2016. Information on mortality was obtained by cross-referencing the NHIRD with the Taiwan National Register of Deaths. The NHIRD is maintained by the Health and Welfare Data Science Center, Ministry of Health and Welfare, Taiwan, and the anonymized data has been made available for research purposes by formal application. Our study was approved by the Research Ethics Committee of Hualien Tzu Chi Hospital (REC No: IRB107-152-C); the requirement for informed consent was waived due to the retrospective use of anonymized data. This study was conducted in accordance with the World Medical Association Declaration of Helsinki.

### Study population

We conducted the observational study with target trial emulation to strengthen causal inference [23, 24]; the details of how we emulated a target trial are described in Additional file 1: Table S1. We applied similar selection criteria to those of the target trial to include all adults aged  ≥ 65 years with diagnoses of both AF and DM who had been treated with oral anticoagulants between 2012 and 2019 in NHIRD. The ICD-9-CM code 427.31 and ICD-10-CM codes I48.0, I48.1, I48.2, and I48.91 were used to identify AF diagnosis; the ICD-9-CM code 250 or ICD-10-CM codes E08-E13 were used for DM diagnosis. Both diagnoses should be made at least once in an inpatient service or twice in outpatient services. We restricted our study population to patients aged  ≥ 65 years because both AF and HF developed mainly in older adults.

We excluded patients without AF and DM diagnoses at baseline. We excluded patients with end-stage renal disease (ESRD), rheumatic heart disease, congenital heart disease, or having valve replacement surgery before the index date because those patients are more likely to receive warfarin over NOACs [25], and their exclusion helped minimize a potential confounding-by-indication bias. To apply the new-user design, those with a prescription of any oral anticoagulants in 2011 were excluded, enhancing the likelihood of identifying new oral anticoagulant users since 2012 when NOACs were introduced in Taiwan’s National Health Insurance program. We excluded those with index dates in 2020, ensuring at least 1-year follow-up for each patient. Finally, we excluded patients with any prior HF diagnoses before the index date (Additional file 1: Figure S1).

### Exposures, outcomes, and follow-up

To emulate a target trial with intention-to-treat analysis, we used an as-started design that divided patients into NOAC and warfarin groups according to their first oral anticoagulant use regardless of subsequent prescriptions [24]. The index date (time zero of follow-up) was defined as the date of initiation of oral anticoagulant treatment, and follow-up began since then.

The primary outcome was the incident HF diagnosed in an inpatient service or at least three times in an outpatient service (ICD-9-CM code: 428; ICD-10-CM code: I50). The date of the first HF diagnosis was assigned as the date of event occurrence. We followed up with each patient from their index date until an occurrence of the outcome event, death, or December 31, 2020 (the last date in our database), whichever came first.

In our main analyses, we compared HF risk between overall NOACs versus warfarin. We further performed subgroup analyses that subclassified NOACs into four subgroups (dabigatran, rivaroxaban, apixaban, and edoxaban) and compared each with warfarin. We also performed analyses stratified by age (65–74 and  ≥ 75 years), sex, and hospital levels.

### Covariates and confounders

Pre-existing comorbidity was defined as a condition diagnosed at least once on an inpatient basis or twice on an outpatient basis within the year prior to the index date. The Charlson comorbidity index was calculated to quantify the overall comorbidity status [26]. We also calculated the CHA2DS2-VASc score, which is used to predict stroke risk and determine whether an oral anticoagulant should be used in clinical practice [27, 28]. We defined baseline antidiabetic drugs based on the treatment prescribed within 1 month prior to the index date; the number of diabetes medication types were also calculated. Other baseline medications were defined as a drug prescribed for at least 30 days within the year prior to the index date. The duration of AF and DM were defined as the period from the date of first diagnosis of AF or DM to the date of initiating oral anticoagulants (index date). The index year, monthly income (derived from income-related insurance premiums), physician’s medical specialty, and the hospital level of oral anticoagulant initiation were also retrieved as covariates [17].

### Propensity score-based fine stratification weighting

We calculated the propensity score for each patient to estimate the probability of initiating NOACs using multivariable logistic regression models, including all covariates shown in Table 1. We used fine stratification weights based on propensity scores to create more exchangeable groups with balanced characteristics for comparisons. Two fine stratification weighting methods were applied to cover both targets of inference: estimation of the average treatment effect in the whole population (ATE) and estimation of the average treatment effect among the treated population (ATT) [29]. The individuals were stratified into 50 strata by the propensity score distribution; how the weights were calculated in each stratum is described elsewhere [29]. The propensity score-based fine stratification weighting was conducted individually for each comparison set, including that of overall analyses, subgroup analyses, stratified analyses, or sensitivity analyses.

**Table 1:** Baseline characteristics of older patients with atrial fibrillation and diabetes receiving NOAC or warfarin after propensity score-based fine stratification weighting

	Population with fine stratification weights (ATE)^*^			Population with fine stratification weights (ATT)^**^		
	NOAC (N = 19,591)	Warfarin (N = 5,117)	SMD^†^	NOAC (N = 19,591)	Warfarin (N = 5,117)	SMD^†^
Age (years)^‡^	76.6 ± 7.3	76.7 ± 7.6	0.013	76.8 ± 7.4	76.9 ± 7.7	0.013
Sex						
Male	52.3	53.0	0.014	52.3	53.6	0.026
Female	47.7	47.0	0.014	47.7	46.4	0.026
Charlson comorbidity index^‡§^	2.7 ± 2.0	2.7 ± 1.9	0.000	2.7 ± 2.0	2.6 ± 1.9	0.051
CHA2DS2-VASc score^‡#^	4.3 ± 1.5	4.2 ± 1.5	0.067	4.2 ± 1.5	4.1 ± 1.6	0.065
Comorbidities						
Hypertension	78.1	77.1	0.024	77.5	76.3	0.029
Coronary artery disease	30.5	30.6	0.002	30.0	30.2	0.004
COPD	12.9	13.6	0.021	12.7	13.6	0.027
Chronic kidney disease	13.7	14.3	0.017	13.2	14.1	0.026
Cirrhosis	4.0	4.5	0.025	3.5	4.2	0.036
Hyperlipidemia	39.8	39.3	0.010	40.3	39.8	0.010
Stroke	32.2	29.9	0.050	32.0	28.6	0.074
Rheumatoid arthritis	0.8	0.7	0.012	0.8	0.7	0.012
Gout	9.6	9.5	0.003	9.2	9.1	0.004
Dementia	7.3	6.8	0.020	7.6	6.9	0.027
Malignancy	9.4	9.3	0.003	9.5	9.4	0.003
Medication use						
Statins	40.2	39.7	0.010	41.3	40.8	0.010
ACEI or ARB	61.7	61.8	0.002	61.9	62.0	0.002
β blockers	44.5	45.8	0.026	44.3	45.9	0.032
Calcium channel blockers	46.9	47.4	0.010	45.9	46.6	0.014
Diuretics	22.3	23.7	0.033	21.0	23.0	0.048
NSAID	33.4	32.6	0.017	33.4	32.3	0.023
Corticosteroids	5.8	5.7	0.004	5.7	5.6	0.004
Antipsychotics	5.3	5.2	0.005	5.1	5.1	0.000
Proton pump inhibitors	9.0	8.2	0.029	9.1	8.0	0.039
Baseline diabetes medications						
Metformin	48.6	47.4	0.024	49.3	47.5	0.036
Sulfonylurea	31.4	30.4	0.022	30.1	28.9	0.026
Meglitinide	5.7	5.5	0.009	5.2	5.0	0.009
AGI	7.7	8.7	0.037	7.3	8.6	0.048
TZD	4.8	4.6	0.010	4.8	4.4	0.019
DPP-4i	26.7	27.7	0.023	27.6	29.1	0.033
SGLT-2i	1.7	2.0	0.022	2.1	2.4	0.020
GLP-1 RA	0.2	0.2	0.000	0.2	0.2	0.000
Insulin	10.2	10.0	0.007	9.9	9.8	0.003
Numbers of diabetes medications						
Without medications	33.3	33.8	0.011	33.7	34.5	0.017
1 type	24.4	23.3	0.026	24.2	22.8	0.033
2 types	21.4	22.4	0.024	21.3	22.0	0.017
≥ 3 types	20.9	20.5	0.010	20.8	20.7	0.003
Duration of diabetes^&^						
< 2 years	21.8	20.6	0.029	18.0	15.8	0.059
≥ 2 years	78.2	79.4	0.029	82.0	84.2	0.059
Duration of AF^&^						
< 2 years	71.2	68.5	0.059	69.9	66.0	0.084
≥ 2 years	28.8	31.5	0.059	30.1	34.0	0.084
Index year						
2012–2013	13.3	13.5	0.006	6.5	6.6	0.004
2014–2015	23.6	24.0	0.009	21.2	21.7	0.012
2016–2017	30.4	28.9	0.033	33.9	32.1	0.038
2018–2019	32.7	33.6	0.019	38.4	39.5	0.023
Income level (NTD)						
Financially dependent	29.5	29.2	0.007	29.5	29.1	0.009
15,840–29,999	47.8	47.9	0.002	47.2	47.6	0.008
30,000–44,999	11.2	11.1	0.003	11.2	11.0	0.006
≥ 45,000	11.6	11.7	0.003	12.2	12.2	0.000
Hospital level of OAC initiation						
Medical center	36.2	34.1	0.044	38.2	35.2	0.062
Regional hospital	44.7	47.0	0.046	44.4	47.3	0.058
District hospital or clinic	19.1	19.0	0.003	17.5	17.5	0.000
Physician specialty						
Cardiologist	63.7	66.2	0.052	65.2	68.5	0.070
Neurologist	19.5	16.7	0.073	20.3	16.4	0.101
Others	16.8	17.2	0.011	14.5	15.0	0.014

Data are presented as percentages unless otherwise noted

*ACEI* angiotensin-converting enzyme inhibitors, *AF* atrial fibrillation, *AGI* alpha-glucosidase inhibitors, *ARB* angiotensin II receptor blockers, *ATE* average treatment effect in the whole population, *ATT* average treatment effect among the treated population, *COPD* chronic obstructive pulmonary disease, *DPP-4i* dipeptidyl peptidase-4 inhibitors, *GLP-1 RA* glucagon-like peptide-1 receptor agonists, *IPTW* inverse probability of treatment weighting, *NOAC* non-vitamin K antagonist oral anticoagulant, *NSAID* nonsteroidal anti-inflammatory drugs, *NTD* New Taiwan Dollar, *OAC* oral anticoagulant, *PSM* propensity score matching, *SGLT-2i* sodium-glucose cotransporter-2 inhibitors, *SMD* standardized mean difference, *TZD* thiazolidinedione

^*^The pseudo-population constructed by propensity score-based fine stratification weighting to estimate the average treatment effect in the whole population

^**^The pseudo-population constructed by propensity score-based fine stratification weighting to estimate the average treatment effect among the treated population

^†^A standardized mean difference of  < 0.1 indicates a negligible difference

^‡^Presented as mean ± standard deviation

^§^Calculated without scores for age

^#^Congestive heart failure, hypertension, age  ≥ 75 years, diabetes mellitus, stroke or transient ischemic attack, vascular disease, age 65–74 years, sex category (CHA2DS2-VASc) score

^&^The period from the date of first diagnosis of diabetes or AF to the index date

### Statistical analyses

The difference in baseline characteristics was determined by standardized difference, with a value of  < 0.1 considered negligible. The standardized difference is preferred to significance testing of covariates between study groups because it is not confounded by sample sizes or the statistical power [30]. We estimated the cumulative incidences and cause-specific hazard ratios (HRs) of HF using cause-specific Cox proportional hazard models with death treated as a censoring event [31]. To address the potential cluster effect and variation from each different hospital or clinic (where oral anticoagulant treatment was initiated), we included shared frailty, estimating the cluster random effect of hospital/clinic, into the regression model [32, 33]. A two-tailed probability (p) value < 0.05 was considered statistically significant. We managed data and performed statistical analyses using SAS software, version 9.4 (SAS Institute, Inc., Cary, NC, USA) and STATA, version 15 (Stata Corporation LLC, College Station, TX, USA).

### Sensitivity analyses

Various sensitivity analyses were conducted to determine the robustness of our study results. First, to consider the treatment adherence during follow-up, we applied the on-treatment design (analog of per-protocol design in clinical trials) in which the follow-up would be censored when the oral anticoagulant type was switched or discontinued. Discontinuation was defined as patients without a refilled prescription of the index oral anticoagulant 90 days after the last prescription. Second, we restricted our analysis to those taking the index oral anticoagulant with a high medication possession ratio, defined as  ≥ 80%. The medication possession ratio was calculated by dividing the number of days with prescription of oral anticoagulants by the days of the follow-up period [34]. Third, we excluded patients with any diagnoses of chronic kidney disease (CKD) before the index date since we could not obtain individuals’ renal function data, which may influence the choice of NOACs. Fourth, to determine whether potential variations between physicians who initiated the NOAC or warfarin prescription influenced our results, we performed a sensitivity analysis that included shared frailty, estimating the cluster random effect of different physicians, into the regression model. Additionally, we performed two sensitivity analyses with different statistical designs. One sensitivity analysis applied propensity score matching (rather than fine stratification) was performed to balance patient characteristics between groups; the matching was based on the nearest-neighbor matching algorithm without replacement, with a caliper width equal to 0.2 standard deviation of the logit of the propensity score [35, 36]. Another sensitivity analysis estimated the adjusted HRs by multivariable Cox regression models based on the original cohort without applying propensity score methods.

## Results

### Patient characteristics

We initially included 24,835 patients (19,710 NOAC and 5,125 warfarin users) after applying the inclusion and exclusion criteria; the patient characteristics in the original cohort are shown in Additional file 1: Table S2. For further analyses, we constructed pseudo-populations containing 19,591 NOAC and 5,117 warfarin users after applying propensity score-based fine stratification weighting. The patient characteristics in the weighted population for ATE and ATT estimation are presented in Table 1. The mean age was approximately 76.6 years, and female patients accounted for 47% of all participants. The mean follow-up duration was 3.0 years. Patient characteristics were balanced appropriately between groups after fine stratification weighting, with standardized differences  < 0.1. The flowchart of patient selection is presented in Additional file 1: Figure S1.

### Risk of incident HF

**Fig. 1:**
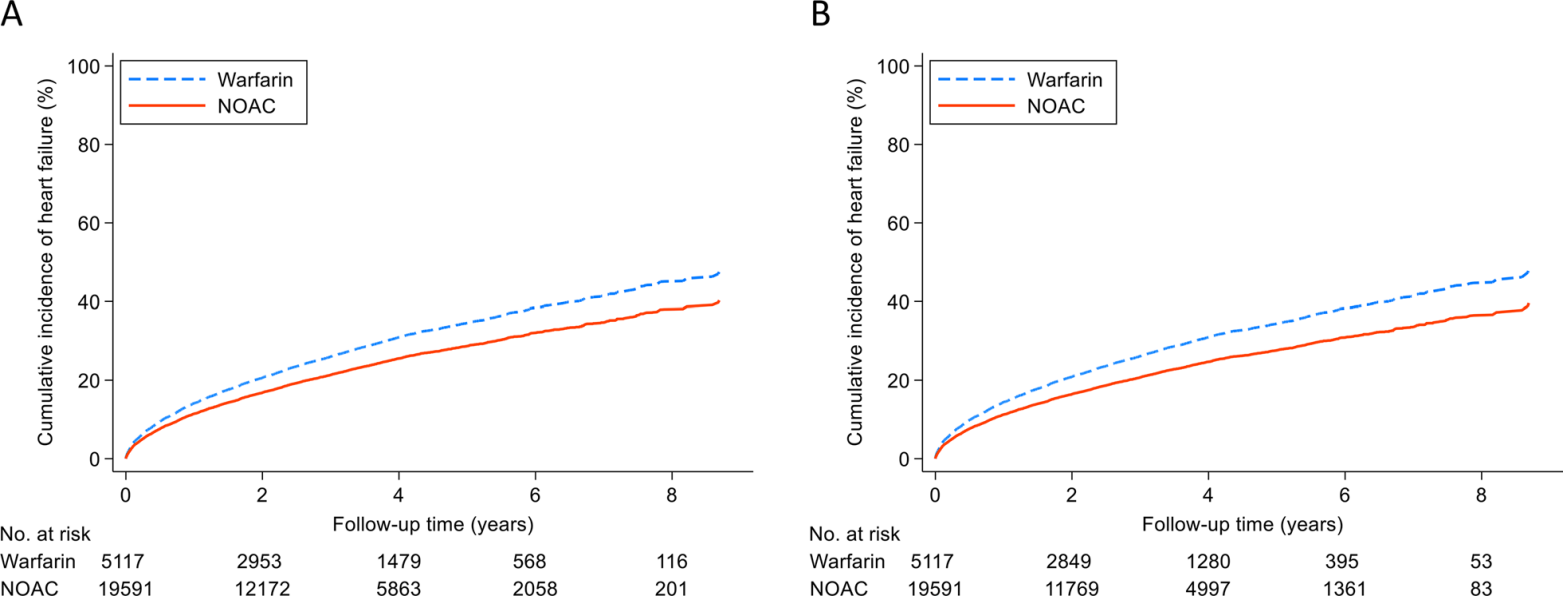
The cumulative incidence curves of HF in elderly patients with AF and DM taking NOACs and those taking warfarin. The curves were estimated according to the pseudo-populations constructed by **A** propensity score-based fine stratification weighting estimating ATE and **B** that estimating ATT. *AF* atrial fibrillation, *ATE* average treatment effect in the whole population, *ATT* average treatment effect among the treated population, *DM* diabetes mellitus, *NOAC* non-vitamin K antagonist oral anticoagulant, *HF* heart failure

In the analysis with propensity score-based fine stratification weighting for ATE estimation, NOAC use was significantly associated with a lower risk of developing HF than warfarin use (HR = 0.80, 95% confidence interval CI 0.74–0.86, p < 0.001). In the ATT estimation analysis, a similar result of lower HF risk in NOAC users was observed (HR = 0.77, 95% CI 0.70–0.84, p < 0.001) (Table 2). Figure 1 illustrates the curves for cumulative HF incidences in patients taking NOACs and those taking warfarin; a lower cumulative HF incidence was observed in NOAC users. The curves for estimating ATE and ATT are shown in Fig. 1A and B, respectively.

**Table 2:** Risk of heart failure in older patients with atrial fibrillation and diabetes receiving NOAC versus warfarin

	Event no	Person-years	Incidence rate^†^	HR (95% CI)	p-value
Fine stratification weights estimating ATE^*^					
NOAC (N = 19,591)	4512	59,298	76.1	0.80 (0.74–0.86)	< 0.001
Warfarin (N = 5,117)	1404	14,677	95.6	1 (ref.)	
Fine stratification weights estimating ATT^**^					
NOAC (N = 19,591)	4158	55,059	75.5	0.77 (0.70–0.84)	< 0.001
Warfarin (N = 5,117)	1343	13,576	98.9	1 (ref.)	

*ATE* average treatment effect in the whole population, *ATT* average treatment effect among the treated population, *CI* confidence interval, *HR* hazard ratio, *NOAC* non-vitamin K antagonist oral anticoagulant, *ref.* reference

^*^Propensity score-based fine stratification weighting which estimated the average treatment effect in the whole population

^**^Propensity score-based fine stratification weighting which estimated the average treatment effect among the treated population

^†^Incidence rate, per 1000 person-years

In the ATE estimation analyses for each NOAC, dabigatran (HR = 0.86, 95% CI 0.80–0.93, p < 0.001), rivaroxaban (HR = 0.80, 95% CI 0.74–0.86, p < 0.001), apixaban (HR = 0.78, 95% CI 0.68–0.90, p < 0.001), and edoxaban (HR = 0.72, 95% CI 0.60–0.86, p < 0.001) were all associated with a lower HF risk when compared with warfarin (Table 3). The ATT estimation analyses demonstrated similar findings.

**Table 3:** Risk of heart failure in older patients with atrial fibrillation and diabetes receiving each NOAC versus warfarin

	Fine stratification weights estimating ATE^*^		Fine stratification weights estimating ATT^**^	
	HR^†^ (95% CI)	p-value	HR^†^ (95% CI)	p-value
Dabigatran vs. warfarin	0.86 (0.80–0.93)	< 0.001	0.81 (0.75–0.88)	< 0.001
Rivaroxaban vs. warfarin	0.80 (0.74–0.86)	< 0.001	0.77 (0.71–0.83)	< 0.001
Apixaban vs. warfarin	0.78 (0.68–0.90)	< 0.001	0.72 (0.62–0.83)	< 0.001
Edoxaban vs. warfarin	0.72 (0.60–0.86)	< 0.001	0.66 (0.54–0.81)	< 0.001

*ATE* average treatment effect in the whole population, *ATT* average treatment effect among the treated population, *CI* confidence interval, *HR* hazard ratio

^*^Propensity score-based fine stratification weighting which estimated the average treatment effect in the whole population

^**^Propensity score-based fine stratification weighting which estimated the average treatment effect among the treated population

^†^The HR is calculated using patients taking warfarin as the reference group

In the analyses stratified by age, sex, and hospital levels, consistent findings were observed; the significantly lower HF risk associated with NOAC use was observed in all stratified groups, regardless of age, sex, or hospital levels (Table 4).

**Table 4:** Risk of heart failure in older patients with atrial fibrillation and diabetes receiving NOAC versus warfarin, stratified for age, sex, and hospital levels

	Fine stratification weights estimating ATE^*^		Fine stratification weights estimating ATT^**^	
	HR^†^ (95% CI)	p-value	HR^†^ (95% CI)	p-value
Age				
65–74 years	0.79 (0.68–0.92)	0.003	0.75 (0.62–0.90)	0.002
≥ 75 years	0.80 (0.71–0.90)	< 0.001	0.77 (0.67–0.89)	< 0.001
Sex				
Male	0.71 (0.63–0.81)	< 0.001	0.67 (0.57–0.78)	< 0.001
Female	0.86 (0.78–0.96)	0.009	0.85 (0.75–0.96)	0.007
Hospital level				
Medical center	0.83 (0.73–0.94)	0.003	0.81 (0.70–0.93)	0.003
Regional hospital	0.84 (0.75–0.94)	0.002	0.81 (0.71–0.93)	0.002
District hospital or clinic	0.78 (0.65–0.94)	0.009	0.76 (0.61–0.94)	0.011

*ATE* average treatment effect in the whole population, *ATT* average treatment effect among the treated population, *CI* confidence interval, *HR* hazard ratio, *NOAC* non-vitamin K antagonist oral anticoagulant, *ref.* reference

^*^Propensity score-based fine stratification weighting which estimated the average treatment effect in the whole population

^**^Propensity score-based fine stratification weighting which estimated the average treatment effect among the treated population

^†^The HR is calculated using patients taking warfarin as the reference group

### Results of sensitivity analyses

With the application of an on-treatment design, NOAC users still demonstrated a lower HF risk than warfarin users (HR = 0.67, 95% CI 0.60–0.75, p < 0.001) in the ATE estimation analysis (Table 5). In the analysis restricted to only patients with a high medication possession ratio (≥ 80%), a more remarkable association between NOAC use and lower HF risk was observed (HR = 0.47, 95% CI 0.40–0.56, p < 0.001) (Table 5). In the analysis that excluded patients with CKD, a similar result of a lower HF risk in NOAC users was observed (HR = 0.79, 95% CI 0.72–0.87, p < 0.001) (Table 5). Additionally, the analysis including shared frailty to address the potential cluster random effect of different physicians also demonstrated a similar result (HR = 0.80, 95% CI 0.74–0.86, p < 0.001). The above sensitivity analyses for ATT estimation demonstrated consistent results. In the analysis applying propensity score matching or using multivariable regression models to adjust for covariates without propensity score methods, NOAC users still had a lower HF risk than warfarin users (Additional file 1: Table S3). The baseline patient characteristics in the analysis applying propensity score matching are shown in Additional file 1: Table S4; the patient characteristics in the analysis using multivariable regression models only are shown in Additional file 1: Table S2. Overall, all the sensitivity analyses generated comparable results as our primary analyses, further supporting the robustness of our findings.

**Table 5:** Risk of heart failure in older patients with atrial fibrillation and diabetes receiving NOAC versus warfarin in the sensitivity analysis applying on-treatment design, that restricting patients with MPR  ≥ 80%, that excluding patients with CKD, and that considering cluster effects of different physicians

	Fine stratification weights estimating ATE^*^		Fine stratification weights estimating ATT^**^	
	HR^†^ (95% CI)	p-value	HR^†^ (95% CI)	p-value
Applying on-treatment design				
NOAC vs warfarin	0.67 (0.60–0.75)	< 0.001	0.64 (0.57–0.72)	< 0.001
Restricting on patients with MPR ≥ 80%				
NOAC vs warfarin	0.47 (0.40–0.56)	< 0.001	0.45 (0.38–0.55)	< 0.001
Excluding patients with CKD				
NOAC vs warfarin	0.79 (0.72–0.87)	< 0.001	0.76 (0.69–0.85)	< 0.001
Considering cluster effects of different physicians^‡^				
NOAC vs warfarin	0.80 (0.74–0.86)	< 0.001	0.77 (0.70–0.84)	< 0.001

*ATE* average treatment effect in the whole population, *ATT* average treatment effect among the treated population, *CI* confidence interval, *CKD* chronic kidney disease, *HR* hazard ratio, *MPR* medication possession ratio, *NOAC* non-vitamin K antagonist oral anticoagulant, *ref.* reference

^*^Propensity score-based fine stratification weighting which estimated the average treatment effect in the whole population

^**^Propensity score-based fine stratification weighting which estimated the average treatment effect among the treated population

^†^The HR is calculated using patients taking warfarin as the reference group

^‡^We included shared frailty, estimating the cluster random effect of different physicians, into the regression model to consider the potential variation from each different physician who initiated the NOAC/warfarin prescription

## Discussion

This nationwide retrospective cohort study demonstrated that elderly adults with AF and DM taking NOACs had an approximately 20% lower risk of incident HF than those taking warfarin. The association between NOAC use and decreased HF risk was consistent, regardless of age, sex, hospital-level subgroups, or the estimations for ATE or ATT. The findings were further supported by several sensitivity analyses. Notably, the lower risk of HF associated with NOAC use versus warfarin use was more remarkable in patients taking oral anticoagulants with a high medication possession ratio and when applying the on-treatment design to the analysis, implying the robust association between oral anticoagulant choices and HF risk.

Although the exact mechanisms of lower HF risk in NOAC users could not be determined in our study, several hypotheses could help explain our findings. Previous preclinical evidence has suggested that both factor Xa and thrombin have activities beyond coagulation, including involvement in inflammation, atherosclerotic plaque progression, atherothrombosis, vascular remodeling, and tissue fibrosis [18–20]. Among NOACs, rivaroxaban, apixaban, and edoxaban are factor Xa inhibitors, and dabigatran is a direct thrombin inhibitor; the inhibition of factor Xa or thrombin theoretically not only affects the function of coagulation but also the aforementioned activities. Recent preclinical and clinical studies have further supported that NOACs have potential anti-inflammatory effects, reduce atherosclerosis, help prevent ischemic heart disease, and suppress the progression of cardiac fibrosis and ischemic cardiomyopathy [18–20, 37, 38], all of which may restrain the pathophysiology of cardiac dysfunction and HF, further decreasing the risk of developing HF. In addition, previous studies have indicated that poor diabetes control increases the risk of developing HF [5, 39, 40]. Hyperglycemia, insulin resistance, and hyperinsulinemia could trigger a cascade of deleterious effects, such as inflammation, dyslipidemia, endothelial dysfunction, activation of the renin–angiotensin–aldosterone system, autonomic dysfunction, and cardiac fibrosis, which further cause both ischemic cardiomyopathy and diabetic cardiomyopathy, predisposing HF development [5]. Previous studies have found a beneficial role of vitamin K in improving insulin sensitivity and glucose tolerance and reducing insulin resistance through several mechanisms [41–43]. In recent real-world studies, better blood glucose and diabetes control were suggested in patients taking NOACs than in those taking warfarin due to the presence or absence of their mechanisms of antagonizing vitamin K [15–17]. It is therefore plausible to support that one of the explanations for NOACs being associated with lower HF risk than warfarin may be via their beneficial effects on glycemic and diabetes control.

Some existing studies have evaluated the efficacy and safety of NOACs versus those of warfarin for stroke prevention in AF patients already coexisting with HF [44, 45]. However, to our knowledge, evidence regarding the risk of incident HF in those treated with NOACs versus those treated with warfarin is still lacking. Our study focused on elderly AF patients with DM, a vulnerable population prone to HF, and demonstrated that NOACs were associated with a decreased risk of incident HF compared with warfarin. Such findings have important clinical impacts because HF coexisting with AF and DM could increase the risk of stroke, for which oral anticoagulants are mainly prescribed for prevention, and substantially deteriorate patient prognosis and quality of life [5, 10, 11]. Our results suggested that NOACs are the preferred oral anticoagulant treatment among elderly AF patients with DM when considering the prevention of HF development in this vulnerable population.

The main strengths of our study were the use of a real-world nationwide database representing Taiwan’s entire population, the target trial emulation design strengthening causal inference using observational data, the novel findings demonstrating the different risks of HF between different oral anticoagulant users, and the study robustness supported by various sensitivity analyses. However, some limitations should be acknowledged. First, we could not gather data on lifestyle, smoking and drinking history, and detailed laboratory examination results (e.g., blood glucose and renal function). Additionally, the indication for which the physicians had chosen warfarin over NOAC (or vice versa) for each patient could not be obtained from the claims-based dataset. Although we employed propensity score methods (including fine stratification weighting and matching) and multivariable regressions to exclude potential confounders, there may still be some unknown or unmeasured confounders. Second, we were unable to access patients’ comprehensive medical records to confirm diagnostic accuracy due to the patient anonymity policy in the NHIRD; therefore, potential misclassification errors may exist in the claims-based data. However, misclassifications among patients taking NOACs and those taking warfarin are non-differential, thereby pushing the estimates towards the null [46, 47]. Since we already observed a significant difference in the HF risk between NOACs and warfarin in our study, the true effect sizes may be larger than we observed. Third, some patients could alter the types of oral anticoagulants used during follow-up; hence, our main analysis with an as-started design (emulating intention-to-treat analysis) may underestimate the true effect sizes for differences in HF risk between NOAC and warfarin groups. In the sensitivity analysis with an on-treatment design (analog of per-protocol) and that limited to patients with a high medication possession ratio of index anticoagulant treatment, we further obtained larger effect sizes with more significant results. Such results implied that our findings of lower HF risk in NOAC users might be genuine and merits further confirmation in future studies. Fourth, our study focused on a vulnerable population, namely elderly patients with AF and DM; patient baseline characteristics revealed a significant comorbidity status in our study population. However, it remains unclear whether the observed lower HF risk among NOAC users can be generalized to younger or healthier patients; more research is required to answer this question.

## Conclusions

In this nationwide retrospective cohort study, elderly patients with AF and DM taking NOACs had a lower risk of incident HF than those taking warfarin. Our findings suggest that NOACs may be the preferred oral anticoagulant treatment to reduce the risk of HF in elderly AF patients with DM. Future research is warranted to elucidate causation and investigate the underlying mechanisms of our findings.

## Supplementary Information

**Additional file 1: Figure S1. **Flowchart of patient selection. **Table S1.** Specification and emulation of a target trial evaluating the effect of NOACs versus warfarin on the risk of incident heart failure using real-world data from Taiwan’s NHIRD. **Table S2.** Baseline characteristics of elderly patients with atrial fibrillation and diabetes receiving NOAC or warfarin in the original population, without weighting or matching. **Table S3.** Risk of heart failure in elderly patients with atrial fibrillation and diabetes receiving NOAC versus warfarin in the sensitivity analysis applying propensity score matching and that applying multivariable regression models without propensity score methods. **Table S4.** Baseline characteristics of elderly patients with atrial fibrillation and diabetes receiving NOAC or warfarin in the population after propensity score matching.

## Data Availability

The dataset used in this study is managed by the Taiwan Ministry of Health and Welfare and thus cannot be made available publicly. Researchers interested in accessing this dataset can submit a formal application to the Ministry of Health and Welfare to request access (Taiwan Ministry of Health and Welfare, No. 488, Sect. 6, Zhongxiao E Rd, Nangang District, Taipei 115, Taiwan; website: https://dep.mohw.gov.tw/DOS/cp-2516-59203-113.html).

## Abbreviations

HF: Heart failure
AF: Atrial fibrillation
DM: Diabetes mellitus
NOAC: Non-vitamin K antagonist oral anticoagulant
HR: Hazard ratio
CI: Confidence interval
NHIRD: National Health Insurance Research Database
ESRD: End-stage renal disease
ATE: Average treatment effect in the whole population
ATT: Average treatment effect in the treated population
CKD: Chronic kidney disease
