# Supplementary material for: Risk of heart failure in elderly patients with atrial fibrillation and diabetes taking different oral anticoagulants: a nationwide cohort study
Source: Cardiovasc Diabetol. 2023 Jan 6;22:1. doi: 10.1186/s12933-022-01688-1 (PMC9824984; doi:10.1186/s12933-022-01688-1)
Supplement: Supplementary file 1 — Additional file 1: Figure S1. Flowchart of patient selection. Table S1. Specification and emulation of a target trial evaluating the effect of NOACs versus warfarin on the risk of incident heart failure using real-world data from Taiwan’s NHIRD. Table S2. Baseline characteristics of elderly patients with atrial fibrillation and diabetes receiving NOAC or warfarin in the original population, without weighting or matching. Table S3. Risk of heart failure in elderly patients with atrial fibrillation and diabetes receiving NOAC versus warfarin in the sensitivity analysis applying propensity score matching and that applying multivariable regression models without propensity score methods. Table S4. Baseline characteristics of elderly patients with atrial fibrillation and diabetes receiving NOAC or warfarin in the population after propensity score matching. [file 12933_2022_1688_MOESM1_ESM.pdf]

Supplemental Materials

Risk of heart failure in elderly patients with atrial fibrillation and diabetes taking different oral anticoagulants: A nationwide cohort study

Figure S1. Flowchart of patient selection

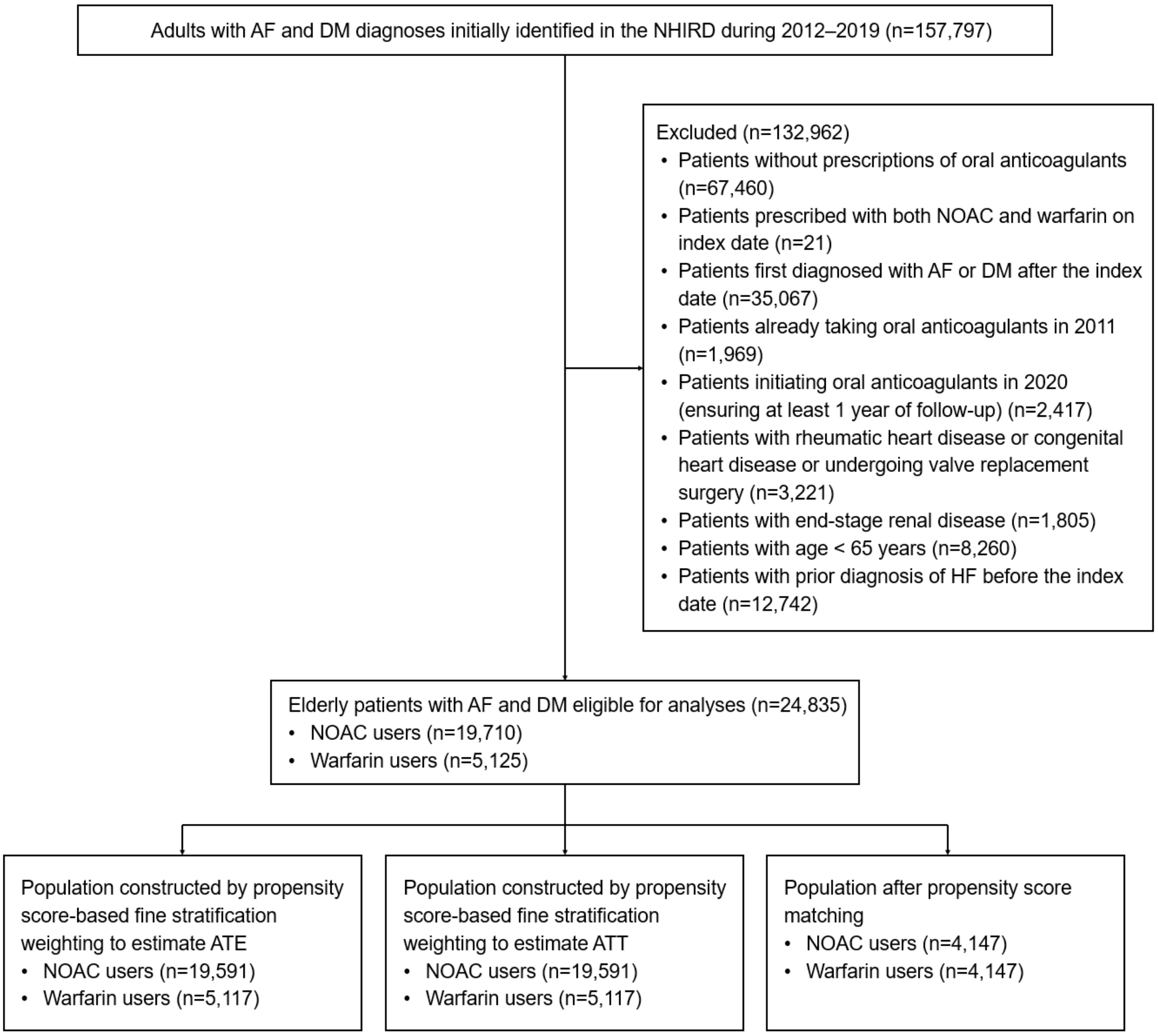

Abbreviations: AF, atrial fibrillation; ATE, average treatment effect in the whole population; ATT, average treatment effect among the treated population; DM, diabetes mellitus; NHIRD, National Health Insurance Research Database; NOAC, non-vitamin K antagonist oral anticoagulant

Table S1. Specification and emulation of a target trial evaluating the effect of NOACs versus warfarin on the risk of incident heart failure using real-world data from Taiwan's NHIRD

Component	Target trial	Trial emulation using real-world data
Aim	To investigate the effect of NOAC versus warfarin on the risk of incident HF in elderly patients with AF and DM	Same
Eligibility	Older adults aged ≥65 years previously diagnosed with AF and DM without severe valvular heart disease or end-stage renal disease<br>New users of oral anticoagulants	Same, but excluding patients with a diagnosis of rheumatic heart disease, congenital heart disease, or having valve replacement surgery, since severe valvular heart disease is hard to directly define using claims data in NHIRD.
Treatment strategies	NOAC and warfarin	Same
Treatment assignment	Eligible patients are randomly assigned to NOAC and warfarin groups.	Using propensity score methods (e.g., propensity score-based fine stratification weighting or propensity score matching) to construct a study population with a similar probability of treatment assignment between NOAC and warfarin groups.
Follow-up	Follow-up begins at treatment assignment and ends at occurrence of HF, death, loss to follow-up, or on 31 December 2020, whichever occurs first	Same (in a real-world scenario, the treatment assignment and initiation occur at the same time)
Outcome	Incident HF	Same
Causal contrast	Primary analysis: intention-to-treat effect (effect of being assigned to NOAC versus warfarin at baseline, regardless of whether patients continue following the assigned treatment after baseline)	Same (modified as as-started design, an analog of intention-to-treat)
	Sensitivity analysis: per-protocol effect (effect of following the treatment strategies in the study	Same (modified as on-treatment design, an analog of per-protocol)

	protocol at baseline and after baseline)	
Statistical analysis	Cox proportional hazards models to estimate cumulative incidences and hazard ratios	Same

Abbreviations: AF, atrial fibrillation; DM, diabetes mellitus; HR, heart failure; NHIRD, National Health Insurance Research Database; NOAC, non-vitamin K antagonist oral anticoagulant.

Table S2. Baseline characteristics of elderly patients with atrial fibrillation and diabetes receiving NOAC or warfarin in the original population, without weighting or matching.

	Original population*		
	NOAC (N = 19,710)	Warfarin (N = 5,125)	SMD†
Age (years)‡	76.8 ± 7.4	76.2 ± 7.1	0.083
Sex			
Male	52.4	51.0	0.028
Female	47.6	49.0	0.028
Charlson comorbidity index‡§	2.7 ± 2.0	2.8 ± 2.0	0.050
CHA2DS2-VASc score‡#	4.2 ± 1.5	4.4 ± 1.5	0.133
Comorbidities			
Hypertension	77.5	80.0	0.061
Coronary artery disease	30.0	32.3	0.050
COPD	12.7	13.5	0.024
Chronic kidney disease	13.1	15.3	0.063
Cirrhosis	3.5	5.9	0.114
Hyperlipidemia	40.4	37.4	0.062
Stroke	32.1	34.7	0.055
Rheumatoid arthritis	0.8	0.7	0.012
Gout	9.1	10.9	0.060
Dementia	7.6	6.5	0.043
Malignancy	9.5	9.0	0.017
Medication use			
Statins	41.4	35.6	0.119
ACEI or ARB	62.1	60.8	0.027
β blockers	44.4	45.5	0.022
Calcium channel blockers	45.9	50.7	0.096
Diuretics	20.9	26.3	0.127
NSAID	33.4	33.6	0.004
Corticosteroids	5.7	6.2	0.021
Antipsychotics	5.1	5.7	0.027
Proton pump inhibitors	9.0	9.0	0.000
Baseline diabetes medications			
Metformin	49.5	46.8	0.054
Sulfonylurea	30.0	36.5	0.138
Meglitinide	5.2	7.4	0.091
AGI	7.3	8.9	0.059
TZD	4.8	5.2	0.018
DPP-4i	27.8	22.2	0.130
SGLT-2i	2.3	0.5	0.154

GLP-1 RA	0.2	0.2	0.000
Insulin	9.9	10.8	0.030
Numbers of diabetes medications			
Without medications	33.5	31.1	0.051
1 type	24.2	25.2	0.023
2 types	21.4	23.9	0.060
≥ 3 types	20.8	19.7	0.027
Duration of diabetes[&]			
< 2 years	17.9	39.4	0.490
≥ 2 years	82.1	60.6	0.490
Duration of AF[&]			
< 2 years	69.7	77.9	0.187
≥ 2 years	30.3	22.1	0.187
Index year			
2012–2013	6.5	39.8	0.859
2014–2015	21.0	32.7	0.266
2016–2017	33.7	16.5	0.405
2018–2019	38.8	11.0	0.679
Income level (NTD)			
Financially dependent	29.5	29.6	0.002
15,840–29,999	47.1	49.1	0.040
30,000–44,999	11.2	11.4	0.006
≥ 45,000	12.2	9.8	0.077
Hospital level of OAC initiation			
Medical center	38.5	29.8	0.184
Regional hospital	44.1	45.5	0.028
District hospital or clinic	17.3	24.8	0.185
Physician specialty			
Cardiologist	65.2	57.0	0.169
Neurologist	20.4	17.7	0.069
Others	14.4	25.4	0.278

Data are presented as percentages unless otherwise noted.

[*]All eligible patients were included, without reconstructing the population by any propensity score methods.

[†]A standardized mean difference of <0.1 indicates a negligible difference.

[‡]Presented as mean ± standard deviation.

[§]Calculated without scores for age.

[#]Congestive heart failure, hypertension, age ≥ 75 years, diabetes mellitus, stroke or transient ischemic attack, vascular disease, age 65 to 74 years, sex category (CHA2DS2-VASc) score

[&]The period from the date of first diagnosis of diabetes or AF to the index date.

Abbreviations: ACEI, angiotensin-converting enzyme inhibitors; AF, atrial fibrillation; AGI, alpha-glucosidase inhibitors; ARB, angiotensin II receptor blockers; ATE, average treatment effect in the whole population; ATT,

average treatment effect among the treated population; COPD, chronic obstructive pulmonary disease; DPP-4i, dipeptidyl peptidase-4 inhibitors; GLP-1 RA, glucagon-like peptide-1 receptor agonists; IPTW, inverse probability of treatment weighting; NOAC, non-vitamin K antagonist oral anticoagulant; NSAID, nonsteroidal anti-inflammatory drugs; NTD, New Taiwan Dollar; OAC, oral anticoagulant; PSM, propensity score matching; SGLT-2i, sodium-glucose cotransporter-2 inhibitors; SMD, standardized mean difference; TZD, thiazolidinedione

Table S3. Risk of heart failure in elderly patients with atrial fibrillation and diabetes receiving NOAC versus warfarin in the sensitivity analysis applying propensity score matching and that applying multivariable regression models without propensity score methods

	Event no.	Person-years	Incidence rate†	HR (95% CI)	p-value
Propensity score matching*					
NOAC (N = 4,147)	1157	15209	76.1	0.85 (0.79–0.93)	<0.001
Warfarin (N = 4,147)	1286	14439	89.1	1 (ref.)	
Multivariable regression model**					
NOAC (N = 19,710)	4,171	55,261	75.5	0.85 (0.80–0.91)	<0.001
Warfarin (N = 5,125)	1,641	18,908	86.8	1 (ref.)	

*Analysis was conducted in the population after propensity score matching.

**Analysis was conducted based on the original population; the multivariable regression model was used to adjust all covariates listed in Table 1.

†Incidence rate, per 1000 person-years.

Abbreviations: CI, confidence interval; HR, hazard ratio; NOAC, non-vitamin K antagonist oral anticoagulant; ref., reference.

Table S4. Baseline characteristics of elderly patients with atrial fibrillation and diabetes receiving NOAC or warfarin in the population after propensity score matching

	Population after propensity score matching*		
	NOAC (N = 4,147)	Warfarin (N = 4,147)	SMD†
Age (years)‡	76.5 ± 7.1	76.3 ± 7.3	0.028
Sex			
Male	52.0	51.7	0.006
Female	48.0	48.3	0.006
Charlson comorbidity index‡§	2.7 ± 2.0	2.8 ± 2.0	0.050
CHA2DS2-VASc score‡#	4.4 ± 1.5	4.3 ± 1.5	0.067
Comorbidities			
Hypertension	79.3	79.3	0.000
Coronary artery disease	31.8	32.0	0.004
COPD	13.4	13.0	0.012
Chronic kidney disease	14.6	15.5	0.025
Cirrhosis	5.4	5.3	0.004
Hyperlipidemia	37.8	37.7	0.002
Stroke	33.3	31.3	0.043
Rheumatoid arthritis	0.7	0.8	0.012
Gout	10.3	10.8	0.016
Dementia	6.4	6.3	0.004
Malignancy	8.6	9.3	0.025
Medication use			
Statins	37.4	36.4	0.021
ACEI or ARB	63.4	61.0	0.050
β blockers	45.4	44.8	0.012
Calcium channel blockers	49.9	49.3	0.012
Diuretics	25.3	25.8	0.012
NSAID	34.4	33.8	0.013
Corticosteroids	5.4	5.8	0.017
Antipsychotics	5.0	5.6	0.027
Proton pump inhibitors	9.0	9.2	0.007
Baseline diabetes medications			
Metformin	47.7	46.0	0.034
Sulfonylurea	35.0	35.1	0.002
Meglitinide	6.5	6.9	0.016
AGI	8.9	8.8	0.004
TZD	4.7	4.8	0.005
DPP-4i	24.7	23.8	0.021
SGLT-2i	0.9	0.6	0.035

GLP-1 RA	0.2	0.2	0.000
Insulin	10.7	11.2	0.016
Numbers of diabetes medications			
Without medications	31.8	32.0	0.004
1 type	25.1	24.6	0.012
2 types	22.3	23.5	0.029
≥ 3 types	20.9	19.9	0.025
Duration of diabetes[&]			
< 2 years	27.2	28.1	0.020
≥ 2 years	72.8	71.9	0.020
Duration of AF[&]			
< 2 years	71.4	73.3	0.043
≥ 2 years	28.6	26.7	0.043
Index year			
2012–2013	26.4	26.4	0.000
2014–2015	39.9	39.9	0.000
2016–2017	20.3	20.3	0.000
2018–2019	13.5	13.5	0.000
Income level (NTD)			
Financially dependent	29.9	29.7	0.004
15,840–29,999	48.9	49.2	0.006
30,000–44,999	10.8	10.9	0.003
≥ 45,000	10.5	10.2	0.010
Hospital level of OAC initiation			
Medical center	34.9	31.4	0.074
Regional hospital	44.3	44.5	0.004
District hospital or clinic	20.9	24.0	0.074
Physician specialty			
Cardiologist	62.1	60.5	0.033
Neurologist	17.9	16.0	0.051
Others	20.0	23.5	0.085

Data are presented as percentages unless otherwise noted.

[*]The population included after propensity score matching.

[†]A standardized mean difference of <0.1 indicates a negligible difference.

[‡]Presented as mean ± standard deviation.

[§]Calculated without scores for age.

[#]Congestive heart failure, hypertension, age ≥ 75 years, diabetes mellitus, stroke or transient ischemic attack, vascular disease, age 65 to 74 years, sex category (CHA2DS2-VASc) score

[&]The period from the date of first diagnosis of diabetes or AF to the index date.

Abbreviations: ACEI, angiotensin-converting enzyme inhibitors; AF, atrial fibrillation; AGI, alpha-glucosidase inhibitors; ARB, angiotensin II receptor blockers; ATE, average treatment effect in the whole population; ATT,

average treatment effect among the treated population; COPD, chronic obstructive pulmonary disease; DPP-4i, dipeptidyl peptidase-4 inhibitors; GLP-1 RA, glucagon-like peptide-1 receptor agonists; IPTW, inverse probability of treatment weighting; NOAC, non-vitamin K antagonist oral anticoagulant; NSAID, nonsteroidal anti-inflammatory drugs; NTD, New Taiwan Dollar; OAC, oral anticoagulant; PSM, propensity score matching; SGLT-2i, sodium-glucose cotransporter-2 inhibitors; SMD, standardized mean difference; TZD, thiazolidinedione
